# Discharged. Not Where to Lay His Head

**Published:** 1886-10-16

**Authors:** George Fleming


### DISCHARGED.

### "NOT WHERE TO LAY HIS HEAD."

By George Fleming.

IV.

Lemon had given his bundle in charge of the boy at the newspaper-stall on first getting out at the station. By the time he reached the coffee tavern, of which Mary Parker had spoken, that queer feeling of physical exhaustion, in which everything he touched, the chairs and tables, the very floor he stepped on, seemed deadened as if covered with a thin sheet of cotton-wool,—this odd weariness, which yet was not altogether unpleasant, had fallen upon him once more. His knees shook when he tried to walk. He had some tea, and asked for a room, and he was grateful to the people of the house for accepting without question his statement that he was one of Tillotson's regular workmen called up to the West End for a paying job. It was not true; but a man grows accustomed to a very liberal handling of statement when he has nothing but his own stock of reminiscences to depend upon, with which to enliven workhouse days. It is a curious fact that to shut up a great number of human beings together, in whatever class of life, should have this discouraging effect upon their average veracity; divine truth being, it would seem, but a shy goddess with retrogressive views about competition. And, after all, what Lemon wanted chiefly was a bed to sleep in. It would be a very keen-edged morality, of an altogether superior type, which could have stopped at that moment, with that dull, burning sensation in throat and chest, to consider fine points of accuracy. And there was, I fear, very little of a superior kind about either this man or his experience; the interest of his life, if indeed it possessed any interest, was to be looked for entirely in his relation to average conditions.

He lay in that bed until very late the next morning. He watched his room-mate pack up his tools, and start off for his day's work without exchanging a word with him. There was the letter to Miss Thornhill to be written; he went over each one of its sentences very carefully in his mind, all the time he was dressing. But the long night's rest had done him good. His spirits had risen; and he ordered his belated breakfast quite independently of the fact that his last sixpence had been already paid away for last night's supper.

It was the emptiest hour of a busy morning, between half-past nine and ten. The long, corridor-shaped coffee-room had been swept, and all the marble-topped tables wiped off since the departure of the first lot of regular customers. The mellow October sunshine and the quiet within made a pleasant contrast to the noisy bustle of the street, and Lemon would have liked nothing better than to stay on there indefinitely, sipping his coffee, and watching the heavy omnibuses roll by.

There was, it is true, a young woman behind the counter, but her face was of a forbidding cast, notwithstanding the evident care she had taken to train her hair in ringlets. She was reading a novel, too, and only nodded silently in answer to Lemon's pleasant "Good morning!" She gave him the sheet of paper and the stamped envelope which he asked for, taking them out of a box on the shelf behind her, almost without lifting her eyes from her book. But when at last he rose from his table by the window she got up too, and cast a discriminating eye upon the relics of his breakfast.

"Room, a shillin'. Two coffees, fourpence. Bread; heggs; paper and stamp;—one and tenpence halfpenny," she said indifferently. She put up her hand to adjust her hair, and then looked at the glass which hung opposite to note the effect of her turquoise ring.

"Well, the fact is, Miss ——, I ain't what you might call goin' away just yet. I'm only goin' over to Oxford Street to see about that work o' mine. I've left my kit upstairs; I'm a comin' back, you see, to dinner," Lemon began, rather doubtfully.

"Oh."

She considered for a moment, then raised her voice: "Mother!"

At the second summons a clean-looking middle-aged woman, wearing a checked apron, and with her sleeves rolled up above her elbows, appeared in the inner doorway.

"This man ain't paid for his bed or breakfast. He says he's coming back," the young woman explained languidly.

"What's to pay?"

"One and tenpence ha'penny," she said, and forthwith retired behind her curls and her book.

Lemon repeated his story. He produced his pledge-card this time, and would have made something of his unimpeachable position as a temperance man, but his hostess waived the document somewhat peremptorily aside.

"I don't say nothing for nor yet against it. We're temp'rance people ourselves, as is becomin', seein' 'tis a temp'rance 'ouse. I've seen them cards before now, young man, and I've seen them as swallowed 'em down a deal sight easier than they'd swallow a glass o' water. Them as them pledges hold has got the grip *in* 'em to begin with; and as for the others—I shuts my own cat up pretty tight when I want to keep her nose out o' the milk; that's what *I* say."

She crossed her bare red arms over her substantial waist and stood looking down at him thoughtfully. "You've been ill, I should say, now? I thought as much last night."

"Seven months in hospital. Discharged yesterday. But I'll be in good wages this time to-morrow," Lemon answered eagerly.

"That's accordin'. To-morrow's Sunday. But seven months is a long time. You can leave your things. It won't break us to trust you till to-morrow," the worthy matron added, slowly.

She stepped to the glass-door and watched him striding down the street with Spot at his heels. "Now what's the good to anyone of coddlin' a man up on doctor's stuff for the better part of a year and then turnin' him out like that? Set him up with his pledge! Why can't they have a place for 'em to go to first when they gets discharged? They've got prison missions fast enough. Why don't a man only just off a sick-bed, and full of conceit as an egg is of meat because he's got done with the nursing,—why don't he want looking after for a week or two till he gets steady on his feet like, just as much as if he'd been caught stealin' a handkerchief?—But, Lord, Amelia Jane, one would think to see you that that book was a looking-glass, if so be it you was not born dumb!"

"Do let me alone, mother; I've just got to the place where they're a runnin' off with the di'monds."

"A-ah! you'll sing a different tune, you will, if this here young man runs off with one and tenpence, an' it comes to your father hearin' of it. But, there, I always was a soft one; an' I 'eard 'im cough, cough, in the night. I've been ill myself; and it's hard enough, goodness knows, to keep work a goin' that *is* goin', let alone startin' all things fresh when the life seems clean drained out of your innards. That's what *I* say," the good woman muttered, turning back to her pots and pans.

It was a long walk across the Park and down Oxford Street. When Lemon reached Tillotson's place, his old friend and patron, the foreman, had already gone home. Some of the workmen were putting together their tools, and there was a general air of Saturday afternoon freedom about the look of the workshop, with its piles and rows of half-finished frames. Most of the work was paid for by the piece, and given out to be done at home. There were only a few of the finer workmen kept on the premises, those who copied old designs and could be entrusted with the more delicate carving. These were often changed, many of them being sent for into the country to do fine jobs of restoring.

As Lemon looked about the room now he saw nothing but strange faces, and his heart sank within him. He remembered Miss Thornhill, and he began to feel afraid. Still there was nothing for it but to walk in and take his chance.

"Morning, mates," he began, putting a bold face upon the matter; "can I speak with Mr. Thomas? I hope he ain't gone home yet; he's an old acquaintance of mine."

One or two of the men took their pipes out of their mouths and stared at him in silence. Then a young fellow, who had just sent out for a pot of beer, and was perhaps mollified by the combined pleasures of memory and anticipation, answered civilly enough that Mr. Thomas wasn't there. In point of fact, he had gone home.

"That's bad," said Lemon. "I'm an old hand here myself. I'm only just out of hospital, an' I'm come over to pick up a job," he added, doubtfully.

No one seemed to consider the remark as addressed to himself personally. There was an awkward pause, broken by the young fellow who had already spoken, and who now came to the front again with generous offers of refreshment.

"Here, have a pull at this after your walk,—for luck! I've been in the hospital myself. Broke my leg once, all along of larkin' on a Bank 'Olliday, and a jolly bore it were, to be sure."

He swore with great energy, holding out his pewter pot.

It was months and months since Lemon had seen anything of the kind, and the heady smell of the beer made him turn sick. "No, thank'ee kindly, mate. I'm a temperance man now. Sworn off an' pledged an' all. That's an undoubted fact, an' as such ain't to be—— Why, bless my eyes! if there isn't Old Five Points, an' at the old place too!"

The young workman gazed meditatively into his pot. "Well," he observed philosophically, "the supply of good liquor *bein'* limited, p'raps it's just as well to limit the supply of wise men who drinks it. Here's your good 'ealth, then, matey, an' no malice."

And he turned to watch Lemon making his way across the litter of the workshop towards his unexpected friend.

(*To be continued.*)

